# Supplementary material for: Digital Health Interventions to Improve Access to and Quality of Primary Health Care Services: A Scoping Review
Source: Int J Environ Res Public Health. 2023 Sep 28;20(19):6854. doi: 10.3390/ijerph20196854 (PMC10572344; doi:10.3390/ijerph20196854)
Supplement: Supplementary file 1 [file ijerph-20-06854-s001.zip › ijerph-2548057-supplementary.pdf]

Table S1. Key words employed in the search strategy

**Digital health**	ehealth OR e-health OR "electronic health " OR "digital health " OR "digital technolog* " OR "digital intervention* " OR "electronic care " OR telemedicine OR "tele medicine " OR telehealth OR tele health OR telecare OR tele care OR telemonitoring OR tele monitoring OR teleconsultation OR tele-consultation OR videoconsult* OR "video consult " OR "text messag*" OR texting OR "mobile health " OR "mobile care " OR mhealth OR "m health " OR android OR app OR apps OR audio* OR “cell phone” OR cellphone OR computer* OR mobile OR multi-media OR multimedia OR “personal digital assistant” OR PDA OR SMS OR "social medi*" OR software or telecomm* OR e-Portal OR ePortal OR eTherap* OR e-therap* OR forum* OR “information technolog*” OR "instant messag*" OR internet* OR ipad OR i-pad or iphone OR i-phone OR ipod OR i-pod OR android OR web* OR "smart phone" OR smartphone OR “mobile phone” OR e-mail* OR email*
**UHC**	"Universal Health Care" OR "Health Equity" OR "Health Services Accessibility" OR "Quality of Health Care" OR "health coverage" OR "care coverage" OR "service coverage" OR "treatment coverage" OR "universal coverage" OR "universal health coverage" OR "UHC" OR "financing coverage" OR "Financial risk protection" OR "Financial hardship " OR "Financial protection" OR "Financial protection in health" OR "Efficiency" OR "Equity" OR "Responsiveness" OR "coverage" OR "effectiveness" OR "performance"
**PHC**	"Primary health care" OR "community engagement" OR "intersectoral coordination" OR "multisectoral action*" OR multisectoralism OR "appropriate care" OR "comprehensive health care" OR "Equity" OR "Integrated care" OR "continuity of care" OR resilience

Table S2. Preferred Reporting Items for Systematic reviews and Meta-Analyses extension for Scoping Reviews (PRISMA-ScR) Checklist

SECTION	ITEM	PRISMA-ScR CHECKLIST ITEM	REPORTED ON PAGE #
**TITLE**			
Title	1	Identify the report as a scoping review.	1
**ABSTRACT**			
Structured summary	2	Provide a structured summary that includes (as applicable): background, objectives, eligibility criteria, sources of evidence, charting methods, results, and conclusions that relate to the review questions and objectives.	2
**INTRODUCTION**			
Rationale	3	Describe the rationale for the review in the context of what is already known. Explain why the review questions/objectives lend themselves to a scoping review approach.	3
Objectives	4	Provide an explicit statement of the questions and objectives being addressed with reference to their key elements (e.g., population or participants, concepts, and context) or other relevant key elements used to conceptualize the review questions and/or objectives.	4
**METHODS**			
Protocol and registration	5	Indicate whether a review protocol exists; state if and where it can be accessed (e.g., a Web address); and if available, provide registration information, including the registration number.	4
Eligibility criteria	6	Specify characteristics of the sources of evidence used as eligibility criteria (e.g., years considered, language, and publication status), and provide a rationale.	4
Information sources*	7	Describe all information sources in the search (e.g., databases with dates of coverage and contact with authors to identify additional sources), as well as the date the most recent search was executed.	4, 5
Search	8	Present the full electronic search strategy for at least 1 database, including any limits used, such that it could be repeated.	5
Selection of sources of evidence†	9	State the process for selecting sources of evidence (i.e., screening and eligibility) included in the scoping review.	5
Data charting process‡	10	Describe the methods of charting data from the included sources of evidence (e.g., calibrated forms or forms that have been tested by the team before their use, and whether data charting was done independently or in duplicate) and any processes for obtaining and confirming data from investigators.	5
Data items	11	List and define all variables for which data were sought and any assumptions and simplifications made.	5
Critical appraisal of individual sources of evidence§	12	If done, provide a rationale for conducting a critical appraisal of included sources of evidence; describe the methods used and how this information was used in any data synthesis (if appropriate).	5

SECTION	ITEM	PRISMA-ScR CHECKLIST ITEM	REPORTED ON PAGE #
Synthesis of results	13	Describe the methods of handling and summarizing the data that were charted.	5
**RESULTS**			
Selection of sources of evidence	14	Give numbers of sources of evidence screened, assessed for eligibility, and included in the review, with reasons for exclusions at each stage, ideally using a flow diagram.	5
Characteristics of sources of evidence	15	For each source of evidence, present characteristics for which data were charted and provide the citations.	6
Critical appraisal within sources of evidence	16	If done, present data on critical appraisal of included sources of evidence (see item 12).	6,7
Results of individual sources of evidence	17	For each included source of evidence, present the relevant data that were charted that relate to the review questions and objectives.	6, 7
Synthesis of results	18	Summarize and/or present the charting results as they relate to the review questions and objectives.	7-13
**DISCUSSION**			
Summary of evidence	19	Summarize the main results (including an overview of concepts, themes, and types of evidence available), link to the review questions and objectives, and consider the relevance to key groups.	14
Limitations	20	Discuss the limitations of the scoping review process.	14, 15
Conclusions	21	Provide a general interpretation of the results with respect to the review questions and objectives, as well as potential implications and/or next steps.	15
**FUNDING**			
Funding	22	Describe sources of funding for the included sources of evidence, as well as sources of funding for the scoping review. Describe the role of the funders of the scoping review.	Title page

JBI = Joanna Briggs Institute; PRISMA-ScR = Preferred Reporting Items for Systematic reviews and Meta-Analyses extension for Scoping Reviews.

* Where *sources of evidence* (see second footnote) are compiled from, such as bibliographic databases, social media platforms, and Web sites.

† A more inclusive/heterogeneous term used to account for the different types of evidence or data sources (e.g., quantitative and/or qualitative research, expert opinion, and policy documents) that may be eligible in a scoping review as opposed to only studies. This is not to be confused with *information sources* (see first footnote).

‡ The frameworks by Arksey and O'Malley (6) and Levac and colleagues (7) and the JBI guidance (4, 5) refer to the process of data extraction in a scoping review as data charting.

§ The process of systematically examining research evidence to assess its validity, results, and relevance before using it to inform a decision. This term is used for items 12 and 19 instead of "risk of bias" (which is more applicable to systematic reviews of interventions) to include and acknowledge the various sources of evidence that may be used in a scoping review (e.g., quantitative and/or qualitative research, expert opinion, and policy document).

From: Tricco AC, Lillie E, Zarin W, O'Brien KK, Colquhoun H, Levac D, et al. PRISMA Extension for Scoping Reviews (PRISMAScR): Checklist and Explanation. Ann Intern Med. 2018;169:467–473. doi: 10.7326/M18-0850.

Table S3. Characteristics of included studies, n = 65

Author, year	Study type	Country	Study aim	Digital health solutions		Main findings and author's conclusions	Outcome
				Type	Area of intervention		
Banks et al 2018 (1)	Qualitative	UK	To evaluate whether an e-consultation system improves the ability of practice staff to manage workload and access.	E-consultation system	Managing workload and patient access.	• Most e-consultations resulted in GPs needing to follow up with a telephone or face-to-face appointment because the e-consultation did not contain sufficient information to inform clinical decision making.<br>• A number of challenges were associated with the technology, including increased workload, difficulties in clinical decision making, and administrative problems generated by a lack of system integration.<br>• E-consultations fell short of providing an effective platform for clinicians to consult with patients and did not justify their financial investment in the system.	Negative
Barron et al 2018 (2)	Mixed methods	South Africa	To examine history, successes and challenges of Mobile health messaging service and helpdesk for South African mothers (MomConnect).	mHealth	Maternal and infant health services	• MomConnect has been scaled rapidly through strong government stewardship and leverage of existing technology, content and partnerships.	
Barron et al 2014 (3)	Mixed methods	South Africa		mHealth	Maternal and infant health services	• MomConnect resulted in improvements in the quality of services, e.g. decreased drug stock-outs and change of behaviour of some health workers.	
Casey et al 2017 (4)	Mixed methods	UK	To explore the introduction of one online consultation system (Tele-Doc) and how it shapes working practices.	E-consultation system	PHC service delivery	• Uptake of Tele-Doc by patients was low.<br>• Much of the work of the consultation was redistributed to patients and administrators, sometimes causing misunderstandings.<br>• The 'messiness' of consultations was hard to eliminate.<br>• GPs welcomed varied modes of consulting, but the aspiration of improved efficiency was not realised in practice.	Negative

Coleman et al 2020 (5)	Mixed methods	South Africa	To evaluate the effect of maternal mHealth text messages on uptake of maternal and child health care services in South Africa	mHealth	Maternal and infant health services	• MomConnect resulted in an improvement in achieving complete maternal-infant continuum of care, providing evidence of a positive impact of informative maternal mHealth messages sent to pregnant women and new mothers	
Gonçalves-Bradley et al 2020 (6)	Systematic review	Global	To assess the effects of mobile technologies for supporting communication and consultations between healthcare providers	mHealth	Health workforce performance, healthcare use and patient outcome	• mHealth interventions including a mobile technology may reduce the time between presentation and management of the health condition when primary care providers or emergency physicians use them to consult with specialists.<br>• mHealth interventions may decrease the number of people attending PHC who are referred to secondary or tertiary care in some conditions, such as some skin conditions and CKD.<br>• There was little evidence of effects on participants' health status and well-being, satisfaction, or costs.	Positive – moderate
Agrawal et al 2020 (7)	Systematic review	Global	To assess the effects of digital clinical decision-support systems (CDSS) accessible via mobile devices by primary healthcare providers in the context of primary care settings.	Clinical decision-support systems (CDSS)	Health workforce performance, healthcare use and patient outcome	• It is not clear if decision-support tools used on mobile devices make primary healthcare workers better at following recommended practice.<br>• The evidence is not clear about the effects of these tools on patients' and clients' behaviour and on their health.	Inconclusive
Agarwal et al 2020 (8)	Systematic review	Global	To assess the effects of strategies for notifying stock levels and digital tracking of healthcare-related commodities and inventory via mobile devices across the PHC system	Digital tracking	Stock management and continuity of care	Several factors that may influence the implementation of stock notification and tracking via mobile device. These include:<br>• Challenges tied to infrastructural issues, such as poor access to electricity or internet<br>• Broader health systems issues, such as drug shortages at the national level	Inconclusive
Odendaal et al 2020 (9)	Systematic review	Global	To synthesise qualitative research evidence on health workers' perceptions and experiences of using	mHealth	PHC service delivery	• mHealth changed how health workers worked with each other<br>• mHealth changed how health workers delivered care	Positive

			mHealth technologies to deliver PHC services.			• mHealth led to new forms of engagement and relationships with clients and communities<br>• Health workers' use and perceptions of mHealth could be influenced by factors tied to costs, the health worker, the technology, the health system and society, poor network access, and poor access to electricity	
Dahlgren et al 2021 (10)	Quantitative (secondary analysis)	Sweden	To eExamine determinants for use of direct-to-consumer telemedicine consultations in primary healthcare	Direct-to-consumer (DTC) telemedicine consultations	PHC service delivery	• The factors associated with higher probability of utilization were younger age, higher educational attainment, higher income and being born in Sweden.<br>• The use of DTC telemedicine is determined by factors that are generally not associated with greater healthcare need and the distribution raises some concerns about the equity implications.	NA
Donaghy et al 2019 (11)	Qualitative	UK	To explore patients' and clinicians' experiences of video consultation (VC)	Video consultation	PHC service delivery	• VCs were considered superior to telephone consultations in providing visual cues and reassurance, building rapport, and improving communication.<br>• Technical problems, however, were common. Clinicians felt, for routine use, VCs must be more reliable and seamlessly integrated with appointment systems, which would require upgrading of current IT systems.<br>• When integrated with current systems VCs can provide a time-saving alternative to face-to- face consultations when formal physical examination is not required	NA
Fernemark et al 2020 (12)	Qualitative	Sweden	To investigate primary care physicians' perceived work demands, control over working processes, and social support when providing digital consultation to primary care patients.	Digital consultation	PHC service delivery	• Physicians perceive working with digital consultation as flexible with a high grade of autonomy and reasonable to low demands.<br>• The workload was significantly lower when working with digital consultation.<br>• Offered decisional latitude concerning provider's work situation and allowed for high levels of flexibility in terms of deciding working hours and choosing where to work,	NA
Galles et al 2021 (13)	Quantitative survey	Multiple	To document the experiences with providing telemedicine	Telemedicine	Maternal health service delivery	• Maternal and newborn healthcare providers globally considered telemedicine of benefit during the pandemic and applied it on a wide	NA

			for maternal and newborn healthcare during the pandemic among healthcare professionals globally.			scale for different aspects of maternal and newborn healthcare.<br>• The rapid adaptation to provision of care via telemedicine was not optimally supported by guidelines, training for health providers, adequate equipment, reimbursement for cost of connectivity and insurance payments for care provided remotely.<br>• Healthcare providers worldwide reported not being able to reach a substantial group of families by telemedicine and encountered different barriers in providing high-quality maternity care by telemedicine, and such challenges were more prominent in low-income and middle-income countries.	
Hammersley, et al 2019 (14)	Quasi-experimental method	UK	To explore the content, quality, and patient experience of Video consultation (VC), telephone (TC), and FTFCs in general practice.	Telemedicine	PHC service delivery	• VC may be suitable for simple problems not requiring physical examination.<br>• VC, in terms of consultation length, content, and quality, appeared similar to TC.<br>• Both approaches appeared less 'information rich' than face to face consultation.<br>• Technical problems were common and, though patients really liked VC, infrastructure issues would need to be addressed before the technology and approach can be mainstreamed in primary care.	NA
Hanley et al 2018 (15)	Qualitative	UK	To explore what drove changes to the way telemonitoring was implemented, compare experience of telemonitoring across the range of long-term conditions,	Telemonitoring	PHC service delivery	• Telemonitoring was valued by patients who found it empowering and convenient. This, combined with initial professional concern that increased surveillance may create dependency led to the development of a more patient led service.	
Huygens et al 2017 (16)	Retrospective observational study	Netherlands	To understand the use of email consultation by different patient groups, compared with other general practice (GP) consultations.	Email consultation	PHC service delivery	• Even though email consultation was done in half the general practices in the Netherlands in 2014, the actual use of it is extremely low. Patients who had an email consultation differ from those who had a telephone or face-to-face consultation.<br>• In addition, the use of email consultation by patients is dependent on its provision by GPs	

Jimenez1 et al 2021 (17)	Systematic review	Multiple	to identify the role of digital/health technologies within wider multifeatured interventions that are aimed at enhancing primary care	Unspecified	PHC service delivery	• Digital health resulted in increased patient satisfaction, increased primary care visits compared to specialist visits, and the provision of more health prevention education and improved prescribing practices.<br>• Technologies seem also to increase costs and utilization for some parameters, such as increased consultation costs and increased number of drugs prescribed.<br>• Digital health have not played a major role within comprehensive innovation efforts aimed at enhancing primary care.	
Howells et al 2022 (18)	qualitative study	UK	To explore the experience and impact of organisational and technology changes in response to COVID-19 on access to healthcare for people experiencing homelessness	telephone consultations	PHC service delivery for people experiencing homelessness	The move to remote telephone consultations highlighted the difficulties experienced by participants in accessing healthcare.<br>These barriers including problems at the practice level associated with remote triage as participants did not always have access to a phone or the means to pay for a phone call.<br>The findings have emphasised the importance of addressing practical and technology barriers as well as supporting communication and choice for mode of consultation.	
Kueper et al 2021(19)	Consultation	Canada	To identify priority areas for Artificial Intelligence (AI) and PHC in Ontario, Canada.	AI	Unspecified – PHC	Priorities for artificial intelligence and primary care include provider, patient and system level uses as well as foundational areas related to data and interdisciplinary communication.	
Lall et al 2020 (20)	Systematic review	Multiple	synthesize findings from qualitative or mixed-methods studies to provide insight into factors facilitating or hindering implementation of mLearning strategies for medical and nursing education	mLearning	PHC provider education	The synthesis identified views on valued aspects of mobile devices in terms of efficiency and personalization but concerns over vigilance and poor device functionality; emphasis on the social aspects of technology, especially in a clinical setting; the value of interaction learning for clinical practice; mLearning as a process, including learning how to use a device; and the importance of institutional infrastructure and policies.	
LeFevre et al (21)	Mixed methods	South Africa	To Unpack the performance of a mobile health information messaging program for	mHealth	Maternal and child health	While registration coverage and message delivery success rates exceed those observed for mobile messaging programs elsewhere, study findings highlight opportunities for program improvement	

			mothers (MomConnect) in South Africa:			and reinforce the need for rigorous and continuous monitoring of delivery systems.	
LeFevre et al 2017 (22)	Case study	Ghana	This is case study of the Mobile Technology for Health (MOTECH) program in Ghana, we assess the platform's effectiveness in delivering messages, along with user response across sites in five districts from 2011 to 2014.	mHealth	Unspecified – systems lens	While providers were able to register and upload patient-level health information, the majority of these uploads occurred in Community-based facilities versus Health Centers. For Mobile Midwives, 25% or less of expected messages were received by pregnant women, despite the majority (>77%) owning a private mobile phone. While over 80% of messages received by pregnant women were listened to, postpartum rates of listening declined over time.	
Liaw et al 2021 (23)	Case study	Multiple (HICs)	adaptation, progress, and lessons from four countries with high ICT development regarding primary care informatics response to Covid-19 Pandemic	Digital health, unspecified	Unspecified – systems lens	In countries with high ICT development such as Australia, Canada, United Kingdom and United States, PHC practice transformed and responded rapidly to the COVID pandemic by instituting telehealth and electronic record enabled change. Similar with LMICs, however, disparities in internet access limited adoption in PHC setting located rural and remote areas.	
Liyanage et al 2019 (24)	Delphi study	Multiple	To form consensus about perceptions, issues, and challenges of AI in PHC.	AI	Unspecified – systems lens	PHC and informatics experts reported AI has potential to improve managerial and clinical decisions and processes, and this would be facilitated by common data standards. The respondents did not agree that AI applications should learn and adapt to clinician preferences or behaviour, and they did not agree on the extent of AI potential for harm to patients. It was more difficult to assess the impact of AI-based applications on continuity and coordination of care.	
Lupiáñez-Villanueva et al 2018 (25)	Mixed methods	European Union – multiple	To measure the use of ICT and eHealth applications by GPs in 27 EU member states and analyse the main drivers of and barriers to eHealth adoption in primary healthcare	eHealth	PHC – systems lens	Overall, eHealth adoption in primary healthcare in Europe has increased from 2013 to 2018, but there are differences among the countries surveyed. In countries with the highest level of adoption, the use of eHealth is routine among GPs, while in countries with the lowest level of adoption, eHealth is currently not widespread.	
Mbunge et al 2022 (26)	Systematic review	South Africa	To identify virtual healthcare services and digital health technologies deployed in	eHealth	COVID-19 pandemic response	Although COVID-19 has invigorated the use of digital health technologies, there are still some shortcomings. The authors recommend increasing community networks in rural areas to bridge the	

			South Africa during coronavirus disease 2019 (COVID-19) and the challenges associated with their use.			digital divide and the modification of mHealth policy to advocate for the effective use of innovative technologies in healthcare and the development of sustainable strategies for resources mobilization through private-public partnerships as well as joining available international initiatives advocating for smart digital health.	
Mozes et al 2022 (27)	Mixed methods	Israel	To evaluate attributes and levels of the DCE regarding patients' preferences for telemedicine versus traditional, in-clinic consultation in primary care during the COVID-19 pandemic, in order to facilitate successful implementation.	telemedicine	Primary care post COVID-19	The four most important attributes were: Availability, time until the appointment, severity of the medical problem, patient-physician relationship, and flexible reception hours.	
Murphy et al 2021 (28)	Mixed-methods longitudinal study	UK	To investigate the rapid implementation of remote consulting and explore impact over the initial months of the COVID-19 pandemic.	remote consulting	Primary care during COVID-19	There was universal consensus that remote consulting was necessary.<br>Consultation rates reduced in April to July 2020 compared to 2019.<br>Telephone consulting was sufficient for many patient problems, video consulting was used more rarely, and was less essential as lockdown eased.<br>SMS-messaging increased more than three-fold.<br>GPs were concerned about increased clinical risk and some had difficulties setting thresholds for seeing patients face-to-face as lockdown eased.	
Nadhamuni et al 2021(29)	Framework	India	A framework to drive digital transformation of comprehensive primary health services at scale in India	Digital health	PHC – systems lens	A federated enterprise or platform approach for comprehensive PHC mandates standardisation and security while offering interoperability and customisability. It can scale exponentially by leveraging third-party solutions that can enhance engagement, user experience and efficiency, while relying on consent and privacy frameworks maintained by the platform.	

Neve et al 2020 (30)	Commentary	UK	To analyse risks and recommendation of digital health in PHC	Digital health	PHC – systems lens	Successful adoption of innovative technologies in primary care requires effective preparation of future and current primary care workforce, enabling appropriate use by healthcare professionals, patients, and communities.	
Pagliari et al 2021(31)	Commentary	UK	To reflect on the breadth of digital developments seen in primary care over time, as well as the rapid and significant changes prompted by the COVID-19 crisis	Digital health	PHC – systems lens	Digital transformation in primary care has occurred in incremental steps, but remarkable progress has been achieved over time. Experiences gained during the pandemic have proven useful for driving adoption, altering mindsets and learning lessons, but given the complexities involved in this human-centred discipline, further progress is likely to be gained through an ongoing process of evolution and adaptation rather than revolution and disruption.	
Peters et al 2018 (32)	Commentary	UK	To assess the impact of private online video consulting in primary care	Video consulting	PHC service delivery	Offering rapid, affordable access to primary care advice, private online GP consulting services are expanding	
Peter et al 2018 (33)	Case study	South Africa	ten lessons from MomConnect regarding taking digital health innovation to scale in South Africa	mHealth	Maternal and child health	High-level government buy-in and leadership, complex multistakeholder partnerships, Formal integration with the public health system through facility-based registration, long-term commitment and earmarked funding for core functions were all fundamental to successful implementation of MomConnect in South Africa.	
Rahimi et al 2021 (34)	Systematic scoping review	Multiple	To identify and evaluate published studies that have tested or implemented AI in PHC setting	AI	PHC – systems lens	AIs were primarily implemented for diagnosis, detection, or surveillance purposes. Neural networks (i.e., convolutional neural networks and abductive networks) demonstrated the highest accuracy, considering the given database for the given clinical task.	
Schierhout et al 2020 (35)	Secondary analysis of trial data	India	To explore local variation in the effectiveness of a community health worker managed digital health intervention in rural India	Digital health	PHC – systems lens	Local contextual factors were significant influences on the effectiveness of this DHI-enabled PHC service strategy intervention. Local adaptions need to be planned for, monitored and responded to over time<br>.<br>The findings underscore the importance of exploring and publishing heterogeneity of results, and of conducting flexible process evaluations, to help aid and enrich interpretation of overall trial results.	

Shah et al 2020 (36)	Prospective observational real world feasibility study	UK	to assess an app-based remote patient monitoring solution in reducing the workload of a clinician and reflect this as time-saved in an economic context	remote patient monitoring	PHC provider workload	App-based remote patient monitoring potentially holds large economic benefit to COVID-19 patients. In wake of further waves or future pandemics, and even in routine care, app-based remote monitoring patients could free up vital resources in terms of clinical team's time, allowing a better reallocation of services.	
Shaw et al 2020 (37)	Qualitative study	Multiple	To develop a conceptual practice-based model of eHealth to support health professionals in applying eHealth to their particular professional or discipline contexts	eHealth	Unspecified	Thematic analyses revealed 3 prominent but overlapping domains of eHealth: (1) health in our hands (using eHealth technologies to monitor, track, and inform health), (2) interacting for health (using digital technologies to enable health communication among practitioners and between health professionals and clients or patients), and (3) data enabling health (collecting, managing, and using health data).<br>These domains formed a model of eHealth that addresses the need for clear definitions and a taxonomy of eHealth while acknowledging the fluidity of this area and the strengths of initiatives that span multiple eHealth domains.	
Shieshia et al 2014 (38)	Program evaluation	Malawi	to assess the feasibility, acceptability, and effectiveness of cStock as a mHealth strategy for improving data visibility and reducing stockouts of health products used at the community level	mHealth	Stock management in PHC	Results demonstrate that cStock was feasible and acceptable to test users in Malawi, and that based on comparison with the EPT group, the team component of the EM group was an essential pairing with cStock to achieve the best possible supply chain performance and supply reliability. Establishing multi–level teams serves to connect HSAs with decision makers at higher levels of the health system, align objectives, clarify roles and promote trust and collaboration, thereby promoting country ownership and scalability of a cStock– like system.	
Singh et al 2021 (39)	Mixed methods	India	To explore the factors underpinning scale-up of digital health solutions for front-line health workers (FLHWs) in India, and the potential implications of these factors for sustainability	eHealth	Unspecified	To successfully sustain a scaled up digital tool, it is imperative for all stakeholders, in particular governments and donors, to have an entire supportive ecosystem in place that addresses the dynamics between aspects of the digital solution, actor relationships, implementation processes and key contextual factors, with strong government leadership to align all these pieces.<br>With significant resources spent each year on digital health solutions that are never scaled or	

						sustained, it is imperative that the evidence is first built base on factors that lead to success in sustaining innovations in the digital health space.	
Srinidhi et al 2020 (40)	Case study	India	To describe experience of implementing accredited social health activists (ASHA) Kirana, a digital technology-enabled Maternal Clinical Assessment Tool (M-CAT) and how the ASHAs felt empowered in the process.	mHealth	Unspecified	Well-trained front-line health workers are critical if pregnancy-related risks are to be addressed promptly, especially when women and their families treat these as normal, and antenatal clinics are too crowded for doctors to pay individual attention to every woman.<br><br>Clinical assessment tools powered by digital technologies provide an opportunity to empower front-line workers such as accredited social health activists (ASHAs).	
Turner et al 2022 (41)	Qualitative study	UK	To identify and understand the unintended consequences of online consultations in primary care.	online consultations	Unspecified	Consequences of online consultations were identified that restricted patient access to care by making it difficult for some patients to communicate effectively with a GP and disadvantaging digitally excluded patients.<br>Consequences were identified that limited increases in practice efficiency by creating additional work, isolation, and dissatisfaction for some staff.<br>Unintended consequences often present operational challenges that are foreseeable and partly preventable. Process changes tailored to local circumstances are critical to making effective use of online consultation tools. Unintended consequences also present clinical challenges that result from asynchronous communication.<br>Online consultation tools favour simple, well-formulated information exchange that leads to diffuse relationships and a more transactional style of medicine.	
Ummer et al 2021 (42)	Case study	India	Examine Kerala's use of digital technology during the COVID-19 response	Digital health	COVID – 19 responses	Digital tools in Kerala were able to proliferate rapidly and help meet diverse citizen needs due to high levels of collaboration and intersectoral response that brought together different levels of government and multiple state departments, engaged the private sector, and harnessed the energy of civil society organisations and community volunteers	

Watkins et al 2018 (43)	Qualitative	South Africa	To investigate the use of mobile phones among patients with chronic diseases, pregnant women, and health workers to enhance primary healthcare in rural South Africa	mHealth	PHC – systems lens	The bottom-up use of mobile phones has been evolving to fill the gaps to augment primary care services in South Africa; however, barriers to access remain, such as poor digital infrastructure and low digital literacy.	
WHO global survey 2017 (44)	Quantitative survey	Global	To explore developments in eHealth since the last survey in 2010 and the role it plays in achieving universal health coverage	eHealth	Universal health coverage	There has never been such rapid uptake of any technology as the global spread of mobile communications technology, which has disrupted many established norms.	
Yang et al 2022 (45)	Framework	Unspecified	To propose a framework that identifies five domains for AI/ML integration in primary care to support care delivery transformation	AI / ML	PHC service delivery	PHC plays a critical role in developing, introducing, implementing, and monitoring AI/ML tools in healthcare and must not be overlooked as AI/ML transforms healthcare.	
Yau et al 2019 (46)	Program evaluation	South Africa		an electronic clinical decision support tool	PHC service delivery	The development and implementation of electronic PC101 across four primary care clinics in South Africa has demonstrated its feasibility and has flagged challenges for its further development and scaling.<br>e-PC101 improved quality and delivery of PHC in under-resourced health systems by streamlining the process and providing opportunity to examine clients systematically, comprehensively, and thoroughly.	
Bashshur et al 2016 (47)	Systematic review	Multiple	To present the scientific evidence for the merits of telemedicine interventions in primary care	telemedicine	PHC service delivery	Telemedicine has often been found more acceptable by patients than healthcare providers. Outcomes data are limited but overall suggest that telemedicine interventions are generally at least as effective as traditional care.<br>Telemedicine has significant potential to address many of the challenges facing primary care in today's healthcare environment.	
Farr et al 2018 (48)	Mixed methods	UK	To examine patient and staff views, experiences and acceptability of a UK primary care online consultation system and	e-consultation	PHC service delivery	Most e-consultations resulted in either follow-on phone (32%) or face-to-face appointments (38%) and GPs felt that this duplicated their workload.	

			ask how the system and its implementation may be improved				
Davis et al 2014 (49)	Systematic review	Multiple	To explore the acceptability and feasibility of remote monitoring technology (RMT) use in routine adult patient care, from the perspectives of primary care clinicians, administrators, and clinic staff	Remote monitoring technology	PHC service delivery	Clinicians, staff, and administrators generally held positive views about RMTs.<br>Concerns emerged regarding clinical relevance of RMT data, changing clinical roles and patterns of care, insufficient staffing or time to monitor and discuss RMT data, data incompatibility with a clinic's electronic health record (EHR), and unclear legal liability regarding response protocols.	
Oyeyemi et al 2014 (50)	case–control study	Nigeria	to determine healthcare facility utilization rates in each location as a result to giving cell phones to pregnant women	mHealth	PHC service utilisation	Giving cell phones to pregnant women and generally improving services could increase their utilization of the primary healthcare system.	
Mehl et al 2018 (51)	Case study	South Africa	To examine if mHealth (MomConnect) provides pragmatic starting point for achieving universal health coverage in South Africa	mHealth	Maternal and child health	MomConnect illustrates how an investment in the digital solutions within a particular health programmatic area (e.g., maternal health) can simultaneously contribute towards the development of 'common good' foundational elements of an interoperable digital national health information system.	
Kleij et al 2019 (52)	Opinion piece	Unspecified	To describe Concepts, conditions and challenges of eHealth in primary care.	eHealth	PHC – systems lens	eHealth should support the transition towards personalized medicine, self-management and shared decisions in primary care.<br>Several conditions need to be met to ensure that eHealth applications are safe, evidence-based and of high quality.<br>Innovative but valid research methodology—e.g. adaptive (action research) designs—is a prerequisite for ongoing success and sustainability of eHealth.	
Boers et al 2020 (53)	Opinion piece	Unspecified	To explore the ethical implications of its application in primary care practice	eHealth	PHC – systems lens	The impact of eHealth on primary care is paired with ethical implications including questions of autonomy and professional responsibilities<br>Practice-specific ethical guidance for the use of eHealth in primary care should be developed<br>Primary care professionals should be aware of the ethical implications when using eHealth approaches	

Houwink et al 2020 (54)	Opinion piece	Unspecified	To explore the role of eHealth in health provider education	eHealth	PHC – systems lens	eHealth education should be integrated into vocational training and continuous professional development programmes.<br>Relevant topics are knowledge of applications, impact on stakeholder relationships, data utilisation and digital competence.<br>eHealth training can be delivered in a variety of formats.	
Versluis et al 2020 (55)	Opinion piece	Unspecified	To explore eHealth issues implementation in PHC	eHealth	PHC – systems lens	To successfully implement eHealth in primary care, context-specific implementation strategies are essential.<br>Identifying potential barriers (e.g., costs) and facilitators (e.g., support) to eHealth implementation is necessary to develop the right implementation strategy.<br>The provided tool helps to define the implementation problem and desired implementation behaviour and develop evidence-based implementation strategies.	
Kasteleyn et al 2021 (56)	Opinion piece	Unspecified	To critically appraise of five widely used eHealth applications for PHC	eHealth	PHC – systems lens	eHealth applications show varying degrees of complexity; while all applications generally provide information, additional features may support interaction and in advanced applications data analysis can automate processes.<br>High-risk and high-gain: the higher the complexity, the higher the potential impact.<br>Scientific evidence on effectiveness is often lacking or of insufficient quality.	
Elsyed et al 2020 (57)	Mixed methods	Egypt	To identify factors facilitating or hindering the implementation of EHRs at primary health care (PHC) units as perceived by health care providers (HCPs) at Alexandria city, Egypt.	EHR	PHC – systems lens	The result revealed that "selection of a suitable EHR system, improve information technology system, motivation and incentives of the use and conservation of health care providers time" as the major factors facilitating the successful implementation of EHRs as perceived by health care providers. On the other hand, the main reported barriers hindering the implementation of EHRs are "financial costs, lack of awareness, system maintenance, and resistance to new technologies".	
AlJarullah et al 2018 (58)	Systematic review	Saudi Arabia	To support current policy initiatives by investigating and identifying factors that are likely to affect	Electronic Health Records (EHR)	PHC – systems lens	The authors developed an integrated framework of eight factors that were proven to have a significant direct influence on physicians' acceptance of EHRs: attitude, perceived usefulness, perceived	

			primary care physicians' acceptance of EHRs			ease of use, social influence, computer self-efficacy, perceived threat to physician autonomy, confidentiality concerns, and physician participation	
Ludwick et al 2009 (59)	Systematic review	Multiple	To understand factors and influencers affecting implementation outcomes from previous health information systems implementations experiences.	Electronic Health Records (EHR)	PHC – systems lens	Articles show that systems' graphical user interface design quality, feature functionality, project management, procurement and users' previous experience affect implementation outcomes. Implementers had concerns about factors such as privacy, patient safety, provider/patient relations, staff anxiety, time factors, quality of care, finances, efficiency, and liability.	
				Electronic Health Records (EHR)	PHC – systems lens		

References

1. Banks J, Farr M, Salisbury C, Bernard E, Northstone K, Edwards H, et al. Use of an electronic consultation system in primary care: a qualitative interview study. British Journal of General Practice. 2018;68(666):e1-e8.
2. Barron P, Peter J, LeFevre AE, Sebidi J, Bekker M, Allen R, et al. Mobile health messaging service and helpdesk for South African mothers (MomConnect): history, successes and challenges. BMJ global health. 2018;3(Suppl 2):e000559.
3. Barron P, Pillay Y, Fernandes A, Sebidi J, Allen R. The MomConnect mHealth initiative in South Africa: Early impact on the supply side of MCH services. Journal of public health policy. 2016;37(2):201-12.
4. Casey M, Shaw S, Swinglehurst D. Experiences with online consultation systems in primary care: case study of one early adopter site. British Journal of General Practice. 2017;67(664):e736-e43.
5. Coleman J, Black V, Thorson AE, Eriksen J. Evaluating the effect of maternal mHealth text messages on uptake of maternal and child health care services in South Africa: a multicentre cohort intervention study. Reproductive Health. 2020;17(1):1-9.
6. Gonçalves-Bradley DC, Maria ARJ, Ricci-Cabello I, Villanueva G, Fønhus MS, Glenton C, et al. Mobile technologies to support healthcare provider to healthcare provider communication and management of care. Cochrane Database of Systematic Reviews. 2020(8).
7. Agarwal S, Glenton C, Tamrat T, Henschke N, Maayan N, Fønhus MS, et al. Decision-support tools via mobile devices to improve quality of care in primary healthcare settings. Cochrane Database of Systematic Reviews. 2021(7).
8. Agarwal S, Glenton C, Henschke N, Tamrat T, Bergman H, Fønhus MS, et al. Tracking health commodity inventory and notifying stock levels via mobile devices: a mixed methods systematic review. Cochrane Database of Systematic Reviews. 2020(10).
9. Odendaal WA, Watkins JA, Leon N, Goudge J, Griffiths F, Tomlinson M, et al. Health workers' perceptions and experiences of using mHealth technologies to deliver primary healthcare services: a qualitative evidence synthesis. Cochrane Database of Systematic Reviews. 2020(3).
10. Dahlgren C, Dackehag M, Wändell P, Rehnberg C. Determinants for use of direct-to-consumer telemedicine consultations in primary healthcare—a registry based total population study from Stockholm, Sweden. BMC family practice. 2021;22(1):1-10.
11. Donaghy E, Atherton H, Hammersley V, McNeilly H, Bikker A, Robbins L, et al. Acceptability, benefits, and challenges of video consulting: a qualitative study in primary care. British Journal of General Practice. 2019;69(686):e586-e94.
12. Fernemark H, Skagerström J, Seing I, Ericsson C, Nilsen P. Digital consultations in Swedish primary health care: a qualitative study of physicians' job control, demand and support. BMC family practice. 2020;21(1):1-11.
13. Galle A, Semaan A, Huysmans E, Audet C, Asefa A, Delvaux T, et al. A double-edged sword—telemedicine for maternal care during COVID-19: findings from a global mixed-methods study of healthcare providers. BMJ Global Health. 2021;6(2):e004575.
14. Hammersley V, Donaghy E, Parker R, McNeilly H, Atherton H, Bikker A, et al. Comparing the content and quality of video, telephone, and face-to-face consultations: a non-randomised, quasi-experimental, exploratory study in UK primary care. British Journal of General Practice. 2019;69(686):e595-e604.
15. Hanley J, Pinnock H, Paterson M, McKinstry B. Implementing telemonitoring in primary care: learning from a large qualitative dataset gathered during a series of studies. BMC Family Practice. 2018;19(1):1-11.
16. Huygens MW, Swinkels IC, Verheij RA, Friele RD, van Schayck OC, de Witte LP. Understanding the use of email consultation in primary care using a retrospective observational study with data of Dutch electronic health records. BMJ open. 2018;8(1):e019233.

17. Jimenez G, Matchar D, Koh CHG, van der Kleij R, Chavannes NH, Car J. The role of health technologies in multicomponent primary care interventions: systematic review. Journal of medical Internet research. 2021;23(1):e20195.
18. Howells K, Amp M, Burrows M, Brown J, Brennan R, Dickinson J, et al. Remote primary care during the COVID-19 pandemic for people experiencing homelessness: a qualitative study. British Journal of General Practice. 2022;72(720):e492-e500.
19. Kueper JK, Terry A, Bahniwal R, Meredith L, Beleno R, Brown JB, et al. Connecting artificial intelligence and primary care challenges: findings from a multi stakeholder collaborative consultation. BMJ health & care informatics. 2022;29(1).
20. Lall P, Rees R, Law GCY, Dunleavy G, Cotič Ž, Car J. Influences on the implementation of mobile learning for medical and nursing education: qualitative systematic review by the digital health education collaboration. Journal of medical Internet research. 2019;21(2):e12895.
21. LeFevre AE, Dane P, Copley CJ, Pienaar C, Parsons AN, Engelhard M, et al. Unpacking the performance of a mobile health information messaging program for mothers (MomConnect) in South Africa: evidence on program reach and messaging exposure. BMJ global health. 2018;3(Suppl 2):e000583.
22. LeFevre AE, Mohan D, Hutchful D, Jennings L, Mehl G, Labrique A, et al. Mobile Technology for Community Health in Ghana: what happens when technical functionality threatens the effectiveness of digital health programs? BMC medical informatics and decision making. 2017;17(1):1-17.
23. Liaw S-T, Kuziemsky C, Schreiber R, Jonnagaddala J, Liyanage H, Chittalia A, et al. Primary care informatics response to Covid-19 pandemic: adaptation, progress, and lessons from four countries with high ICT development. Yearbook of medical informatics. 2021;30(01):044-55.
24. Liyanage H, Liaw S-T, Jonnagaddala J, Schreiber R, Kuziemsky C, Terry AL, et al. Artificial intelligence in primary health care: perceptions, issues, and challenges. Yearbook of medical informatics. 2019;28(01):041-6.
25. Francisco Lupiáñez-Villanueva, Frans Folkvord, Clara Faulí. Benchmarking Deployment of eHealth among General Practitioners. 2018.
26. Mbunge E, Batani J, Gaobotse G, Muchemwa B. Virtual healthcare services and digital health technologies deployed during coronavirus disease 2019 (COVID-19) pandemic in South Africa: a systematic review. Global Health Journal. 2022.
27. Mozes I, Mossinson D, Schilder H, Dvir D, Baron-Epel O, Heymann A. Patients' preferences for telemedicine versus in-clinic consultation in primary care during the COVID-19 pandemic. BMC primary care. 2022;23(1):1-10.
28. Murphy M, Scott LJ, Salisbury C, Turner A, Scott A, Denholm R, et al. Implementation of remote consulting in UK primary care following the COVID-19 pandemic: a mixed-methods longitudinal study. British Journal of General Practice. 2021;71(704):e166-e77.
29. Nadhamuni S, John O, Kulkarni M, Nanda E, Venkatraman S, Varma D, et al. Driving digital transformation of comprehensive primary health services at scale in India: an enterprise architecture framework. BMJ global health. 2021;6(Suppl 5):e005242.
30. Neve G, Fyfe M, Hayhoe B, Kumar S. Digital health in primary care: risks and recommendations. British Journal of General Practice. 2020;70(701):609-10.
31. Pagliari C. Digital health and primary care: Past, pandemic and prospects. Journal of global health. 2021;11.
32. Peters L, Greenfield G, Majeed A, Hayhoe B. The impact of private online video consulting in primary care. Journal of the Royal Society of Medicine. 2018;111(5):162-6.
33. Seebregts C, Dane P, Parsons AN, Fogwill T, Rogers D, Bekker M, et al. Designing for scale: optimising the health information system architecture for mobile maternal health messaging in South Africa (MomConnect). BMJ global health. 2018;3(Suppl 2):e000563.

34. Rahimi SA, Légaré F, Sharma G, Archambault P, Zomahoun HTV, Chandavong S, et al. Application of artificial intelligence in community-based primary health care: systematic scoping review and critical appraisal. Journal of medical Internet research. 2021;23(9):e29839.
35. Schierhout G, Praveen D, Patel B, Li Q, Mogulluru K, Ameer MA, et al. Why do strategies to strengthen primary health care succeed in some places and fail in others? Exploring local variation in the effectiveness of a community health worker managed digital health intervention in rural India. BMJ Global Health. 2021;6(Suppl 5):e005003.
36. Shah SS, Safa A, Johal K, Obika D, Valentine S. A prospective observational real world feasibility study assessing the role of app-based remote patient monitoring in reducing primary care clinician workload during the COVID pandemic. BMC Family Practice. 2021;22(1):1-9.
37. Shaw T, McGregor D, Brunner M, Keep M, Janssen A, Barnet S. What is eHealth (6)? Development of a conceptual model for eHealth: qualitative study with key informants. Journal of medical Internet research. 2017;19(10):e8106.
38. Shieshia M, Noel M, Andersson S, Felling B, Alva S, Agarwal S, et al. Strengthening community health supply chain performance through an integrated approach: Using mHealth technology and multilevel teams in Malawi. Journal of global health. 2014;4(2).
39. Singh NS, Scott K, George A, LeFevre AE, Ved R. A tale of 'politics and stars aligning': analysing the sustainability of scaled up digital tools for front-line health workers in India. BMJ Global Health. 2021;6(Suppl 5):e005041.
40. Srinidhi V, Karachiwala B, Iyer A, Reddy B, Mathrani V, Madhiwalla N, et al. ASHA Kirana: when digital technology empowered front-line health workers. BMJ Global Health. 2021;6(Suppl 5):e005039.
41. Turner A, Morris R, Rakhra D, Stevenson F, McDonagh L, Hamilton F, et al. Unintended consequences of online consultations: a qualitative study in UK primary care. British Journal of General Practice. 2022;72(715):e128-e37.
42. Ummer O, Scott K, Mohan D, Chakraborty A, LeFevre AE. Connecting the dots: Kerala's use of digital technology during the COVID-19 response. BMJ Global Health. 2021;6(Suppl 5):e005355.
43. Watkins JOTA, Goudge J, Gómez-Olivé FX, Griffiths F. Mobile phone use among patients and health workers to enhance primary healthcare: A qualitative study in rural South Africa. Social Science & Medicine. 2018;198:139-47.
44. Organization WH. Global diffusion of eHealth: making universal health coverage achievable: report of the third global survey on eHealth: World Health Organization; 2017.
45. Yang Z, Silcox C, Sendak M, Rose S, Rehkopf D, Phillips R, et al., editors. Advancing primary care with Artificial Intelligence and Machine Learning. Healthcare; 2022: Elsevier.
46. Yau M, Timmerman V, Zwarenstein M, Mayers P, Cornick RV, Bateman E, et al. e-PC101: an electronic clinical decision support tool developed in South Africa for primary care in low-income and middle-income countries. BMJ Global Health. 2019;3(Suppl 5):e001093.
47. Bashshur RL, Howell JD, Krupinski EA, Harms KM, Bashshur N, Doarn CR. The empirical foundations of telemedicine interventions in primary care. Telemedicine and e-Health. 2016;22(5):342-75.
48. Farr M, Banks J, Edwards HB, Northstone K, Bernard E, Salisbury C, et al. Implementing online consultations in primary care: a mixed-method evaluation extending normalisation process theory through service co-production. BMJ open. 2018;8(3):e019966.
49. Davis MM, Freeman M, Kaye J, Vuckovic N, Buckley DI. A systematic review of clinician and staff views on the acceptability of incorporating remote monitoring technology into primary care. Telemedicine and e-Health. 2014;20(5):428-38.
50. Oyeyemi SO, Wynn R. Giving cell phones to pregnant women and improving services may increase primary health facility utilization: a case–control study of a Nigerian project. Reproductive health. 2014;11(1):1-8.

51. Mehl GL, Tamrat T, Bhardwaj S, Blaschke S, Labrique A. Digital health vision: could MomConnect provide a pragmatic starting point for achieving universal health coverage in South Africa and elsewhere? BMJ global health. 2018;3(Suppl 2):e000626.
52. van der Kleij RM, Kasteleyn MJ, Meijer E, Bonten TN, Houwink EJ, Teichert M, et al. SERIES: eHealth in primary care. Part 1: Concepts, conditions and challenges. European Journal of General Practice. 2019;25(4):179-89.
53. Boers SN, Jongsma KR, Lucivero F, Aardoom J, Büchner FL, de Vries M, et al. SERIES: eHealth in primary care. Part 2: exploring the ethical implications of its application in primary care practice. European Journal of General Practice. 2020;26(1):26-32.
54. Houwink EJ, Kasteleyn MJ, Alpay L, Pearce C, Butler-Henderson K, Meijer E, et al. SERIES: eHealth in primary care. Part 3: eHealth education in primary care. European Journal of General Practice. 2020;26(1):108-18.
55. Versluis A, van Luenen S, Meijer E, Honkoop PJ, Pinnock H, Mohr DC, et al. SERIES: eHealth in primary care. Part 4: addressing the challenges of implementation. European Journal of General Practice. 2020;26(1):140-5.
56. Kasteleyn MJ, Versluis A, van Peet P, Kirk UB, van Dalfsen J, Meijer E, et al. SERIES: eHealth in primary care. Part 5: A critical appraisal of five widely used eHealth applications for primary care–opportunities and challenges. European Journal of General Practice. 2021;27(1):248-56.
57. Elsyed HMF BN, Abou Hashish EA,. Factors Facilitating or Hindering the Implementation of Electronic Health Records at Primary Health Care Units as Perceived by the Health Care Providers. J Nurs Pract. 2020;3(1).
58. AlJarullah A, Crowder R, Wald M, Wills G. Factors affecting the adoption of EHRs by primary healthcare physicians in the kingdom of Saudi Arabia: an integrated theoretical framework. International Journal of e-Healthcare Information Systems. 2018;5(1):126-38.
59. Ludwick DA, Doucette J. Adopting electronic medical records in primary care: lessons learned from health information systems implementation experience in seven countries. International journal of medical informatics. 2009;78(1):22-31.
